# A non-invasive approach to estimate the energetic requirements of an increasing seabird population in a perturbed marine ecosystem

**DOI:** 10.1038/s41598-018-26647-3

**Published:** 2018-05-29

**Authors:** Davide Gaglio, Richard B. Sherley, Peter G. Ryan, Timothée R. Cook

**Affiliations:** 1 FitzPatrick Institute of African Ornithology, DST-NRF Centre of Excellence, University of Cape Town, Rondebosch, 7701 South Africa; 2 Environment and Sustainability Institute, University of Exeter, Penryn Campus, Penryn, Cornwall, TR10 9FE United Kingdom; 3 Bristol Zoological Society, Clifton, Bristol, BS8 3HA United Kingdom; 4 Institute of Ecology and Environmental Sciences, Evolutionary Eco-physiology Team, University Pierre et Marie Curie, Bât. 44-45, 4ème étage, 4 place Jussieu, 75252 Paris, France

## Abstract

There is a growing desire to integrate the food requirements of predators living in marine ecosystems impacted by humans into sustainable fisheries management. We used non-invasive video-recording, photography and focal observations to build time-energy budget models and to directly estimate the fish mass delivered to chicks by adult greater crested terns *Thalasseus bergii* breeding in the Benguela ecosystem. Mean modelled adult daily food intake increased from 140.9 g·d^−1^ of anchovy *Engraulis capensis* during incubation to 171.7 g·d^−1^ and 189.2 g·d^−1^ when provisioning small and large chicks, respectively. Modelled prey intake expected to be returned to chicks was 58.3 g·d^−1^ (95% credible intervals: 44.9–75.8 g·d^−1^) over the entire growth period. Based on our observations, chicks were fed 19.9 g·d^−1^ (17.2–23.0 g·d^−1^) to 45.1 g·d^−1^ (34.6–58.7 g·d^−1^) of anchovy during early and late provisioning, respectively. Greater crested terns have lower energetic requirements at the individual (range: 15–34%) and population level (range: 1–7%) than the other Benguela endemic seabirds that feed on forage fish. These modest requirements – based on a small body size and low flight costs – coupled with foraging plasticity have allowed greater crested terns to cope with changing prey availability, unlike the other seabirds species using the same exploited prey base.

## Introduction

The balance between energy expenditure and food consumption determines many aspects of animal ecology, including the role of species within ecosystems and the mechanisms that drive population dynamics^1^. As anthropogenic activities and environmental change threaten an increasing number of habitats, there is a growing need to investigate the energy requirements of species dwelling in impacted ecosystems^2–4^ particularly when those species compete with humans for resources^5,6^. Such knowledge can facilitate the development of management plans that account for a species’ needs at the population level.

Accurately measuring energetic needs is particularly important for birds as most species operate at higher trophic levels, exerting top–down control on lower trophic levels and/or reacting to bottom–up forcing^7^. They need regular access to food resources because of their high metabolic rate and energetically demanding flight^8,9^. Birds therefore offer opportunities to explore the relationships between environmental limitations (e.g. climate change), food web characteristics (e.g. trophic relationships) and energy budgets^10^. This requires accurate energetic estimates of individuals in the wild, but these are usually laborious and invasive to obtain. For example, they include the capture of individuals for laboratory work (e.g. surgery, respirometry^11,12^), the use of doubly labelled water^9^ or the deployment of data-loggers^13^. Such methods are becoming a growing ethical concern^14^, particularly for threatened species, making birds a challenging group to study^12,15,16^. Modelling approaches using time-activity budgets combined with knowledge on the energetic costs of specific behaviours offer non-invasive alternatives to estimate bird energy expenditure in the wild^17,18^, and generally provide improved estimates over allometric equations or thermodynamics modelling^18,19^.

Worldwide, many marine environments have been severely altered by human activity with large impacts on top predators^20^. Today ~28% of the world’s ~350 seabird species are considered to be threatened with extinction by the International Union for Conservation of Nature^21^. Moreover, seabirds have high foraging costs and are greatly affected by commercial fishing activities^22–24^. In the North Sea, for example, competition with the industrial fishery for lesser sandeel *Ammodytes marinus* is partly responsible for the low breeding success and population decline of black-legged kittiwakes *Rissa tridactyla* and several other seabird populations^25,26^. Moreover, fluctuations in this key prey appeared to affect disproportionately small, surface-feeding species with high foraging costs, leading to the suggestion that such species – including terns – are sensitive indicators of deterioration in the state of marine ecosystems^27^. Using energetic models to better quantify the consumption of these sensitive seabird species thus offers great potential to integrate their needs into an ecosystem approach to fisheries^18^.

The Benguela ecosystem off southern Africa is one of the four major eastern boundary upwelling ecosystems and one of the most productive ocean areas in the world. Over the last 70 years a combination of fishing and environmental change have altered the availability of lipid-rich forage fish forage in this system, with knock-on consequences for higher trophic level predators^24,28–31^. In particular, the decreased access to prey is considered to be the key driver of ongoing declines of three endemic seabird species: African penguins *Spheniscus demersus*, Cape cormorants *Phalacrocorax capensis* and Cape gannets *Morus capensis*^28–31^. Perhaps surprisingly, numbers of greater crested terns *Thalasseus bergii*, which rely on the same resources and breed in the same region, have tripled over the last few decades; the reasons for these contrasting fortunes remain equivocal^32,33^. Considerable foraging plasticity^34^ and their ability to move breeding sites^35^ could have helped greater crested terns maintain high annual survivorship in the face of ecosystem-wide changes^36^. In addition, it is possible that their small body size (~390 g), single egg clutch, and short breeding period (68 days) reduce the greater crested tern’s overall energy requirements compared to other sympatric breeding seabirds. Thus, estimating energy budgets for the Benguela’s breeding seabirds may help us to understand why numbers of greater crested terns are increasing while the region’s threatened and endemic seabirds that rely on the same resource are decreasing. This information will also improve our knowledge of food partitioning within the Benguela ecosystem food-web, provide a baseline against which to assess the impact of future environmental change, and assist the development of conservation planning.

Here, we report the foraging activity budget of the southern African population of breeding greater crested terns using non-invasive methods. Based on the duration and cost of activities performed by breeding adults, we modelled the daily energy expenditure (DEE) and daily food intake (DFI) of adults during different breeding stages. To account for parameter uncertainty and propagate sources of error, we used Bayesian inference and Markov chain Monte Carlo (MCMC) estimation. We then compared our observed estimates of chick daily food intake to our model results.

## Results

### Time activity budget in relation to breeding stage

Over a total of 51 days, 374 greater crested tern nests were video monitored during incubation and 240 nests during early chick provisioning (hereafter “early provisioning”). These videos provided duration estimates for 1,138 incubation foraging trips and 1,747 early provisioning foraging trips. Over a 16-day period of focal observations, 31 chicks that had left the nest cup (hereafter “mobile chicks”) were monitored during late chick provisioning (hereafter “late provisioning”), which provided duration estimates for 252 foraging trips.

**Figure 1:**
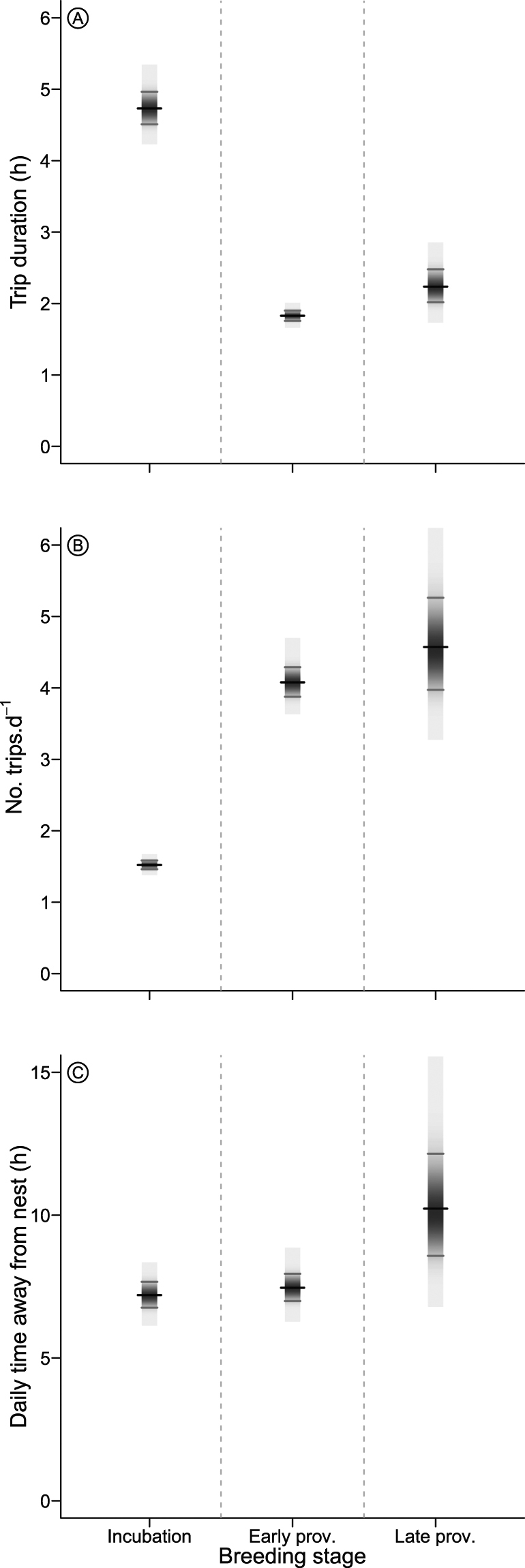
Posterior distributions for foraging effort of greater crested terns breeding at Robben Island (2013–2015) in relation to breeding stage (incubating, early provisioning and late provisioning). (**A**) Daily trip duration, (**B**) number of foraging trips per day, and (**C**) total time spent away from the nest per day for individual greater crested terns. Black tick-marks show means and grey tick-marks 95% Bayesian credible intervals. Prov. = provisioning.

Foraging trips were longer during incubation than during both the early- or late-provisioning periods (Fig. 1A). Incubating adults spent an average of 4.73 h (95% CI 4.51–4.97) away from their nest per trip and performed 1.52 trips·d^−1^ (1.46–1.58, Fig. 1A,B). Foraging trips during early provisioning were shorter (1.83 h, 1.76–1.90), allowing more trips (4.08 trips·d^−1^, 3.88–4.29) than during incubation (Fig. 1B). As a result, the total time spent away from the nest during incubation and early provisioning was similar (Fig. 1C). During late provisioning, when chicks are generally left alone so both adults can forage at once, the mean number of trips per parent per day (4.57 trips·d^−1^, 3.97–5.26) was similar to early provisioning (Fig. 1B). In contrast, the mean duration of each foraging trip was longer (2.24 h, 2.02–2.48), resulting in an increase in the time each parent spent away from the chick (Fig. 1C).

### Modelling time-energy-budgets

**Figure 2:**
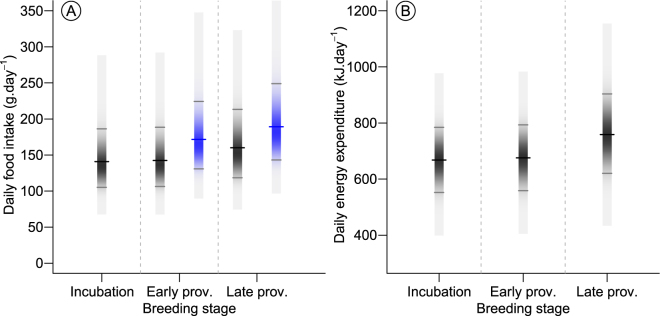
Posterior distributions for (**A**) adult daily food intake (DFI, black bars) and total DFI (single adult DFI + 50% chick DFI, blue bars) related to breeding stage (incubating, early provisioning and late provisioning) for adult greater crested terns provisioning offspring at Robben Island and (**B**) corresponding adult daily energy expenditure. Colour specific tick-marks show means and grey tick-marks 95% Bayesian credible intervals. Prov. = provisioning.

Time-energy budget models indicated that the total energy requirements of adults and offspring increased steadily throughout the breeding season (Fig. 2, Table 1). During incubation, the modelled DEE of an adult was 668 kJ·d^−1^ (95% CI 552–784), with a DFI of 140.8 g·d^−1^ of fish (105.1–186.4, Fig. 2). During early provisioning, adult modelled DEE was 676 kJ·d^−1^ (559–793), which was similar to during incubation. However, the estimated total DFI for an adult, including that fed to the chick, was 22% more at 171.7 g·d^−1^ (130.8–224.3, Fig. 2). During late provisioning, adult modelled DEE increased to 759 kJ·day^−1^ (620–903) with a total modelled DFI, including that of the chick, of 189.2 g·d^−1^ (143.1–248.9, Fig. 2).

**Table 1:** Time-budget and energetic parameters used to model time-energy budgets of greater crested terns in relation to breeding stage (incubating, early chick provisioning and late chick provisioning) and output of these models, including daily energy expenditure (DEE), daily food intake (DFI) and catch per unit effort (CPUE), i.e. the amount of food caught relative to time spent at sea.

Parameter	Incubation	Early provisioning	Late provisioning
**Time (min.day** ^**−1**^ **):**			
- at the colony	1008 ± 14	993 ± 15	826 ± 55
- flying	432 ± 14	447 ± 15	614 ± 55
- diving	1 ± 0.2	1 ± 0.2	1 ± 0.2
Cost resting at colony (kJ·d^−1^)	315.9 ± 28.2	311.0 ± 27.8	259.0 ± 28.6
Cost flying (kJ·d^−1^)	352.1 ± 33.0	364.6 ± 34.3	500.0 ± 62.8
DEE (kJ·d^−1^)	667.9 ± 59.2	675.6 ± 60.0	758.9 ± 72.3
Adult DFI (g·d^−1^)	140.9 ± 20.7	142.5 ± 21.0	160.1 ± 24.2
Chick DFI (g·d^−1^)*	—	29.2 ± 3.9	29.2 ± 3.9
Total DFI (g·d^−1^)	140.9 ± 20.7	171.7 ± 23.9	189.2 ± 27.0
CPUE (g·min^−1^)	0.33 ± 0.05	0.32 ± 0.05	0.26 ± 0.04

Values shown are means ± SD. *Half the daily chick portion, as delivered by one adult and modelled as the mean chick metabolizable energy intake (see Methods).

Using an allometric equation for larids^37^, the modelled mean chick daily metabolizable energy intake was estimated as 358 kJ·d^−1^ (310–405), which results in a chick modelled DFI of 75.6 g·d^−1^ (58.2–98.2 g·d^−1^) over the pre-fledging period. Thus, the expected mean amount returned to chicks across the breeding population – assuming a breeding success of 0.59 chicks fledged per pair – would be 58.3 g·d^−1^ (44.9–75.8 g·d^−1^), or 29.2 g·d^−1^ (22.5–37.9 g·d^−1^) by each parent (Table 1).

Sensitivity analyses showed that variation in adult body mass and prey calorific value had the largest effect on modelled estimates of DFI during all breeding stages (see Supplementary Information S1 and Table S2).

### Estimating chick DFI from photo-sampling, video-recording and focal observations

**Figure 3:**
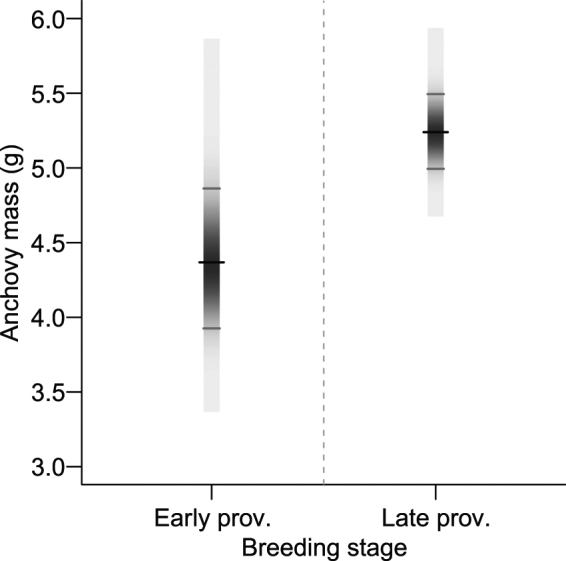
Posterior distributions for mean anchovy mass (g) in the diet of greater crested terns estimated using photo-sampling^34^ across three breeding seasons (2013–2015) at Robben Island during early and late provisioning. Black tick-marks show means and grey tick-marks 95% Bayesian credible intervals. Prov. = provisioning.

The mean (95% CI) mass of anchovies brought to the chick during early provisioning was 4.4 g (3.9–4.9, n = 126), which was smaller than the anchovy returned during late provisioning to mobile chicks (5.2 g; 5.0–5.5, n = 629; Fig. 3). Feeding rates averaged 4.6 fish·d^−1^ (4.1–5.0, n = 240) returned to the nestling during early provisioning, with more fish returned during late provisioning (8.6 fish·d^−1^; 6.6–11.2, n = 34). Chick observed DFI increased from early provisioning (19.9 g·d^−1^, 17.2–23.0, n = 126) to late provisioning (45.1 g·d^−1^, 34.6–58.7, n = 629).

## Discussion

Using a combination of different non-invasive methods, this study presents the first estimates of the time budget and linked energy expenditure of a population of breeding greater crested terns. Our results are in agreement with predictions of central-place foraging models, which indicate that adults should increase the amount of energy delivered to chicks over the chick growth period and so raise their own energy expenditure through increased foraging^13,38^. Small chicks were fed anchovies of a size appropriate to their smaller gape, whereas mobile chicks received anchovies ca 20% heavier. Overall, the amount of fish required daily to feed an adult and chick greater crested tern was 3–7 times lower than for other Benguela endemic species relying on the same prey base (Table 2). A small body size, combined with a highly efficient flight mode and an aptitude for finding food efficiently contribute to lowering the energy budget of greater crested terns. These factors may help to explain why this species’ status remains favourable while populations of other Benguela endemic seabirds relying on the same prey base are decreasing.

**Table 2:** Comparison of population trends, body mass, adult basal metabolic rate (BMR), transport costs and daily food intake (DFI) at individual and population level among four forage fish specialists breeding in the Benguela ecosystem.

Species	G. crested tern	Cape cormorant	Cape gannet	African penguin
IUCN status	Least concern	Endangered	Vulnerable	Endangered
Population trend*	Increasing	Decreasing >50%	Decreasing >30%	Decreasing >50%
Average adult body mass (kg)	0.39	1.2	2.6	3.2
BMR (W.kg^−1^)	6.7	4.9	3.4	3.1
Cost of transport (kJ·kg^−1^·min^−1^)**	2.0	3.9	2.0	1.6
Cost of transport (kJ·min^−1^)**	0.8	4.7	5.3	5.1
Provisioning adult DFI (mean)	187.5 g·d^−1^	547.0 g·d^−1^	1,250 g·d^−1^	758.0 g·d^−1^
(Brood size) Chick DFI (modelled)	(1)~76 g·d^−1^	(2)~210 g·d^−1^	(1)~165 g·d^−1^	(1.5)~330 g·d^−1^
Number of breeding pairs***	~15,000	~190,000	~300,000	~50,000
Breeding population DFI	2,813 kg·d^−1^	103,930 kg·d^−1^	375,000 kg·d^−1^	37,900 kg·d^−1^
**Data sources**	[This study, ^71^]	T. C. *unpubl*.	[^9^,^28^]	[^52^,^77^–^79^]

*Based on the South African Red List citation. **Cost of flight, or swimming in penguins. ***Data from the Department of Environmental Affairs.

### The use of non-invasive methods for assessing energy expenditure

Uncertainties in reconstructing time-energy expenditure can derive from several sources, including the inaccuracy of activity durations^39^, the estimated cost for each behaviour, and thermoregulatory costs. For terns in particular, these parameters may lack precision as energetic investigations on these birds have so far been limited to small numbers of individuals of only a few species^40^. For example, the model used to estimate flight costs may misrepresent energy expenditure compared to more empirical estimates^40–42^. The use of animal-borne data loggers (e.g. GPS, accelerometers) could overcome this limitation, providing precise time-budget data on different at-sea behaviours (e.g. continuous flapping, gliding, hovering and diving) and estimates of their associated energy expenditure^43^. However, we favoured non-invasive methods as animal-borne data loggers can affect bird condition and behaviour^16^, and because greater crested terns are highly sensitive to human disturbance^44^. Furthermore, the approach used in this study can provide better population-level inference than data logger studies, which usually rely on small sample sizes^13,45^.

Observed feeding rates in our study were limited to delivered prey. However, prior to feeding their chick, provisioning adults may be forced to perform specific behaviours which require additional energetic expenditure. Terns are often the target of inter- and intra-specific kleptoparasitism as they bring prey to the colony in their bill^46,47^. This can result in loss of prey (up to 3.2 g·d^−1^ of anchovies for interspecific kleptoparasitism) and/or additional energy costs to counter kleptoparasitic attacks^48^. Accordingly, provisioning adults may have to compensate for the food lost in this way, with implications for their energy expenditure^49^; however, this interaction is poorly understood and few studies can account for the energy expenditure linked to kleptoparasitism in models.

### Implications at the population level of low individual energetic requirements

The recent decreases in seabird populations in the Benguela ecosystem suggest that updated estimates of food consumption are needed to account for energy partitioning in the management of the purse-seine fisheries, with which predators compete for prey^24,31,50^. Modelling approaches are increasingly being implemented to study seabird-fishery competition^23^, including studies to predict the smallest forage fish biomass needed to sustain seabird productivity over the long term^51^. To provide an overview of seabird energetic needs, it is particularly important to account for species body size, clutch size, and number of fledging days. These needs can then be extrapolated to a broader ecosystem level by accounting for the total population breeding in the system.

A comparison of the energetic demands with the other three Benguela endemic seabirds that rely on forage fish, illustrates that the biomass of forage fish needed by breeding greater crested terns at present is much lower than that needed by the other populations (Table 2). Greater crested tern chicks require ~3 kg of anchovy to fledge, compared to ~17 kg of anchovy for an African penguin chick^52^ ~10 kg for a Cape gannet chick^28^ and ~6 kg for a Cape cormorant chick (T. Cook unpublished data). With approximately 15,000 pairs breeding in the Benguela ecosystem, the whole population requires ~2,800 kg·d^−1^ of anchovy, which equates to ~133 times less than the Cape gannet population and ~37 times less than the Cape cormorant population breeding in the region (Table 2). Breeding African penguins, despite a recent decrease in numbers^33^, require ~13 times more food than greater crested terns (Table 2). Thus, their modest energetic requirements may be a key component allowing greater crested terns to cope in a changing and highly exploited environment.

In animals like seabirds, that must travel large distances to secure prey, costs of transport can constitute a large portion of the daily energy budget. Compared to other species of the guild of Benguela ecosystem seabirds specialised on forage fish, the cost of flight per unit of body mass and time in greater crested terns is low (Table 2). Consequently, the overall cost of flight per individual and per time unit in this species is 4–5 times lower than in the other volant seabirds of this guild (Table 2). In part, this can be attributed to their wing morphology. Like other tern species, greater crested terns have long (90–115 cm)^53^, narrow, pointed wings with low wing loading. This makes them efficient at the slow, agile flight needed when searching for food^54^. Terns are capable of rapid turning, swooping, hovering, vertical take-off and soaring^40^, all with relatively low energy expenditure. Their capacity to explore the marine environment efficiently may help explain why greater crested terns appear more successful than the Benguela ecosystem’s other seabird species at coping with decreased food availability.

In the northern Benguela, the population of sardine has been depleted since the early 1970s, and there has been little if any compensation by anchovy, forcing seabirds there to consume low-quality prey such as bearded goby *Sufflogobius bibarbatus*^55^. In contrast to the declining African penguin population, the small population of greater crested terns (~1,200 pairs), which also relies on bearded goby in Namiba^54^, has remained stable, suggesting an ability to cope when switching to low-quality prey^56^. Terns in the North Sea were found to be most vulnerable and sensitive to sandeel exploitation, presumably as a consequence of their specialized diet, small foraging range and inability to increase parental foraging effort when prey becomes scarce^25^. In contrast, greater crested terns breeding in the Benguela ecosystem could buffer these limitations due to their flexible diet, which includes ca. 50 different prey species^34^ and their low fidelity to breeding sites, which are believed to be chosen depending on the local availability of prey immediately preceding the breeding season, rather than by philopatry^32^. In addition, the recent major decrease of migrant tern populations to the Benguela ecosystem (e.g. common tern *Sterna hirundo*^57^) may have led to reduced interspecific competition with surface-gleaning seabirds, providing more resources for this resident tern species. In this context, the greater crested terns’ low energy requirements combined with their ability to switch to alternative prey provide a great advantage, highlighting the apparent species-specific responses to shifting foraging conditions, which seem to favour the greater crested tern in this ecosystem.

In conclusion, this study shows that greater crested terns have relatively low energy requirements at both the individual and population level, when compared to other seabirds breeding in the Benguela ecosystem that rely on the same resources. These low energy requirements appear to contribute to their recent increase in this exploited ecosystem. Further studies implementing detailed knowledge of the energetics, prey demands and demography of the Benguela’s endemic seabirds are needed to understand the apparent differences in their food requirements and assist the development of conservation planning for the threatened seabird species breeding in the region^58,59^.

## Methods

### Measuring time-budget and feeding rates from video-recording and focal observations

Foraging trip durations and offspring feeding rates of breeding greater crested terns were assessed on Robben Island (33°48′S, 18°22′E), in South Africa’s Western Cape Province, using non-invasive video recordings of nest-cup activities during early provisioning (Figure S1). All methods were approved by the Department of Environmental Affairs (RES2013/24, RES2014/83, RES2015/65) and the animal ethics committee of the University of Cape Town (2013/V3/TC).

Greater crested tern chicks become mobile and leave the nest cup after approximately four days^53^. Thus, we monitored individual chicks banded with engraved colour rings using binoculars and a hide (distance 10–30 m) to determine foraging trip durations and feeding rates during late provisioning. Observations and recordings were made from February to May during three breeding seasons (2013, 2014 and 2015). See Supplementary Information S1 for details on these observations.

Video recordings were analysed using VLC media player (VideoLAN project). Three breeding stages were recognised: incubation (during which time, any prey brought to the colony are only used for courtship), early provisioning (the mean week when chicks are provisioned in the nest cup), and late provisioning (the period when adults provision mobile chicks, which typically gather in crèches). Greater crested terns do not forage at night^60^, but our cameras were not always able to capture useable footage from first light or after sunset. Therefore, if birds on focal nests had already left by the start of filming at dawn, or not returned to the nest by the time our cameras could no longer operate due to low light levels, we used nautical twilight as a proxy of their departure and arrival times^61,62^. Nautical twilight is defined when the centre of the sun is 12° below the earth’s horizon^63^. The time of twilight on a given date at each colony was obtained from www.timeanddate.com.

### Estimating chick DFI from photo-sampling

Prey carried by greater crested terns returning to the breeding colony to feed chicks were recorded as part of a program monitoring tern diet^34^. Prey were photographed using a non-invasive photo-sampling technique, allowing for an accurate determination of fish species and standard length^64^ For anchovy, we converted estimated fish lengths to mass using a yearly species-specific regression (see Supporting Information S1 and Table S3).

### Time-energy budget models

Time-energy budget models were built for adult greater crested terns to calculate the amount of food that individuals needed to consume daily to rear their progeny in a season (daily food intake – DFI, g·d^−1^). Specific input values shown in Table 3. Two main behaviours were identified: flying and resting at the colony. Precise time-budget data on at-sea behaviour can be identified using activity recorders such as accelerometers^43^. Due to their small size and sensitivity to disturbance, such data is lacking for almost all tern species. Thus, greater crested terns were assumed to be flying the entire time they were away from the colony. This assumption is supported by the fact that, while foraging, greater crested terns do not rest at the sea surface, diving events are infrequent and dives last only a few seconds at most (pers. obs.). Budgets were based on the bioenergetic model elaborated by Grémillet *et al*.^6^. By considering the duration (*D*) and metabolism per time unit (*M*) of each activity daily energy expenditure (*DEE*, kJ·d^−1^) for adults was defined as:

1 $$DEE=\sum _{k=1}^{n}({D}_{k}\times {M}_{k})$$

*DEE* was then converted into adult DFI. Anchovy make up ~65% of the prey species consumed by greater crested terns in the Western Cape^34^ but since one of our aims was to compare observed estimates of chick DFI to our model results, for the purpose of the model we assume that anchovy makes up the entire diet (but see Supplementary Information S1). Using the mean (±SD) calorific value (*Cp*) of 6.22 ± 0.65 kJ·g^−1^ (wet mass)^65–69^ and an assimilation efficiency^37^ (*Ea*) of 0.77 ± 0.34, we calculated adult DFI (g·d^−1^) as:

2 $$DFI=\frac{DEE}{Cp\times Ea}$$

**Table 3:** Summary of greater crested tern parameters (mean ± SD) and references used to calculate time-energy budgets.

Parameter	Value	Method
Body mass (kg)	0.39 ± 0.03	Measured*
Cost of being at the colony (kJ·kg^−1^·min^−1^)	0.8	Estimated^72^
Cost of flying (kJ·kg^−1^·min^−1^)	2.0	Modelled^73^
Cost of diving (kJ·kg^−1^·min^−1^)	2.0	Modelled^73^
Incubation (days)	28	Measured^53^
Early provisioning (days)	4	Measured^53^
Late provisioning (days)	36	Measured^53^
Fledging (days)	40	Measured^53^
Asymptotic chick mass (g)	370	Modelled^79^
Mean chick MEI (kJ·d^1^)	358.3	Estimated^32,37^
Chicks fledged per pair	0.59	Estimated^70^

*Source = Anthony Tree, pers. comm. BMR = basal metabolic rate. MEI = metabolizable energy intake.

We took adult *DFI* to represent the total energetic needs during each incubation period. For each of the early- and late-provisioning phases, we estimated total adult DFI as the sum of the fish needed to sustain their own expenditure (*DFI*), as derived from their time-activity budget, and the amount needed for chick maintenance and growth. Greater crested tern chicks’ energetic requirements have not been measured before. Chick energetic requirements were thus estimated by fitting an allometric regression to published data on 10 larid species^37^ (Figure S2). This regression yielded a distribution for the total amount of energy metabolized until fledging (*TME*, kJ) in relation to asymptotic chick mass (*A* = 370 g, Table 3):

3 $$TME=\alpha +(\beta \times A)$$

where *α* is the distribution for the estimate of the allometric regression intercept (posterior mean = 539.5) and *β* is the distribution for the estimate of the slope parameter (posterior mean = 37.3). Mean chick daily metabolizable energy intake (*MEI*) (kJ) over the fledging period (40 days) was thus calculated in relation to days taken to fledge (*F*):

4 $$MEI=\frac{TME}{F}$$

We used a breeding success of 0.59 chicks fledged per pair and a fledging period of 40 days^70^ (Table 3) to estimate a daily chick mortality rate (*CMR*) by assuming that nests fail at random through time:

5 $$CMR=\frac{\mathrm{log}(0.59)}{F}$$

We then used the resulting survival function (Figure S3) to estimate total adult DFI (*TDFI*) for each of the early-provisioning (*p* = 1) and late-provisioning (*p = *2) phases as:

6 $$\begin{array}{c}TDF{I}_{p}=DF{I}_{p}+(MEE\times (\frac{{\sum }_{t=1}^{F}\exp (CMR\times t)}{F})\times 0.5),\\ \,\,\,\,\,\,\,\,t=1\ldots F,\,p=1,2\end{array}$$

and estimated *TDFI* across the 40-day fledging period as:

7 $$TDF{I}_{F}=(TDF{I}_{1}\times 0.1)+(TDF{I}_{2}\times 0.9)$$

Metabolic rates of different activities undertaken by the adults were taken from the literature (Table 3). We used a basal metabolic rate (BMR) of 6.73 W kg^−1^ derived from respirometry^71^, 2 × BMR as an estimate of the cost of resting at the colony^72^ and estimated the cost of flying in greater crested terns (as 5.2 × BMR) with the software Flight 1.25^73^ using a wingspan of 1 m^53^, a wing aspect ratio of 10.4 (from the sooty tern *Sterna fuscata*)^73^ and a body mass of 390 g^53^. This software uses aerodynamic modelling, species-specific body mass and dimension to calculate the energetic cost of flying. Terns may use alternative flight modes to continuous flapping (vertical take-off after a dive, hovering over the water in search for prey or gliding) and incur different flight costs depending on the flight mode or the wind field (wind speed and direction). However, we assumed that greater crested terns were flying continuously during their time away from the colony, that the time spent using alternative flight modes was marginal and that overall, greater crested terns experienced an equivalent proportion of different wind speeds and directions. Flight cost (35.6 W·kg^−1^) was thus calculated as the average between the minimum (31.8 W·kg^−1^) and maximum (39.5 W·kg^−1^) power to fly using continuous flapping. Food requirements for the other Benguela endemic seabirds were collected from previous studies (Table 2).

### Statistical analyses

To account for the impact of the uncertainty of the different input parameters on the estimated energy budget, we used MCMC estimation in JAGS (v.4.1.0) via the ‘jagsUI’ library (v. 1.4.2)^74^ for programme R v.3.2.3^75^ to build the time energy budget model. For input parameters (Table 3) where data were normally distributed, we used normal priors with observed means and SDs. Where data were expected to be positive-only with positively-skewed errors (e.g. duration data) we used gamma priors with the observed means for the shape parameter and rate = 1. For the allometric regression between TME and asymptotic chick mass, we used uninformative priors^76^ with *N*(0, 10^−7^) for means (where 10^−7^ is precision) and *U*(1,500, 4,500) for the residual standard error (*σ*), with the precision specified as *σ*^−2^.

To calculate chick DFI estimated from fish mass recorded by photo-sampling, we used the MCMC method described above to fit a gamma regression with a log-link function to estimate the mean (±95% CI) mass of anchovy returned to the colony by breeding stage (early provisioning = 1, late provisioning = 2) from n = 755 photographs. The mean (±95% CI) number of prey delivered to offspring by breeding stage from n = 274 events recorded on video or during focal observations, the mean (±95% CI) foraging trip duration, and the mean (±95% CI) number of offspring feeds per day (feeding rate) by breeding stage (incubation = 1, early provisioning = 2, late provisioning = 3) were also estimated using gamma regressions with a log-link functions. For the gamma regressions, we used uninformative priors, *N*(0, 10^−7^) for the estimated coefficients in the linear predictor and *U*(0, 100) for the shape parameter. The observed chick DFI was calculated by multiplying the posterior distributions for anchovy mass and number of prey delivered.

For all parameters, we modelled means ±95% Bayesian credible intervals (CI) using three MCMC chains (150,000 samples, burn-in of 50,000 and no thinning). All models unambiguously converged (all $\hat{R}$ values < 1.01). See Supporting Information S2 for model code.

## Electronic supplementary material

Supplementary Information
