# Supplementary material for: A non-invasive approach to estimate the energetic requirements of an increasing seabird population in a perturbed marine ecosystem
Source: Sci Rep. 2018 May 29;8:8343. doi: 10.1038/s41598-018-26647-3 (PMC5974355; doi:10.1038/s41598-018-26647-3)
Supplement: Supplementary file 1 — Supplementary Information [file 41598_2018_26647_MOESM1_ESM.docx]

**Supplementary Information**

**Title:** A non-invasive approach to estimate the energetic requirements of an increasing seabird population in a perturbed marine ecosystem

**Authors:** Davide Gaglio, Richard B. Sherley, Peter G. Ryan and Timothée R. Cook

Supplementary Information S1

**Details on field work and bioenergetics modelling:**

**Time-activity budgets and feeding rates:** Video cameras (Sony Camcorder DCR-SX22E) filmed nest-cup activities for 12 hrs·d^-1^ (06h30–18h30, during incubation and early provisioning) to estimate foraging trip duration (time spent away from nest), as well as the average number of trips performed by each parent daily (including trips where prey were lost to kleptoparasitism or not delivered successfully) and daily chick feeding rates (the rate at which fish were successfully delivered by both parents in a day). Filming was carried out on Robben Island at two sub-colonies of different size over 26 days in 2013 and 13 days in 2014, and at one colony over 12 days in 2015 (only one colony established that year). The focal nests were changed on a daily-basis and most video recordings contained a mixture of incubating birds and those undertaking early provisioning in each frame. The ‘single-species’ colony was situated on the north side of the island (33°79’S, 18°36’E) and supported about 7,500 and 8,000 pairs in 2013 and 2014, respectively, but there was no breeding in this area in 2015. The other ‘mixed-species’ colony was an association of terns and Hartlaub’s gulls *Chroicocephalus hartlaubii* established within the human settlement on the island (33°81’S, 18°38’E). This colony totalled ca 2,500, 800 and 8,200 breeding pairs respectively in 2013, 2014 and 2015 (year when the entire island population bred in this area). During both years when there were two colonies on the island (2013 and 2014), colonies were ca 2 km apart. Therefore, with the exception of size, and associations with other breeding species, different colonies were assumed to be under similar environmental conditions (e.g. distance to food patches, influence of wind, tide and temperature).

Video cameras were affixed to tripods and powered via two deep-cycle 12V batteries and an AC/DC power inverter. The whole system was placed 20–50 m from the colony edge and each camera focussed on a group of 6–8 nests that were inside the colony rather than on its edge (Figure S1). Comparable data on trip duration, number of trips and feeding rates was collected from a hide, during late provisioning, using focal observations with binoculars on individual chicks (distance range 10–30 m). At this stage, chicks gather in crèches and are left unattended and both parents forage at sea at the same time^1^. Mobile chicks were followed for as long as possible (range 3.0–9.5 hrs) and trip durations and hourly feeding rates estimates were gathered from observations of chicks, which were followed for at least 3 hrs^2^. During this period, the duration of one trip was defined as the time elapsed between the first and third feeding event observed, and the second feeding event was assumed to represent the start of the alternate parent’s trip. The hourly feeding rates were then multiplied by 12, the maximum daily foraging activity duration for a breeding adult (as chick feeding does not take place after dusk or before dawn^3^) in order to estimate comparable numbers of prey fed to mobile chicks per day. Within these observations, the identification of each focal individual was possible as chicks were banded with metal and engraved colour rings as part of routine ringing operations carried out for the Department of Environmental Affairs (DEA) at the Robben Island colony, or upon a few occasions by the unique characteristics of their plumage.

A single foraging trip was measured, to the nearest second, from the moment a bird left the nest (disappeared from the video frame), until its arrival back at the nest (assuming that for each trip the partner always switched nesting duties). As terns spend little time at the nest when switching with their partner (2.6 ± 3.3 min and 8.2 ± 7.2 min on average during incubation and early provisioning, respectively) and assuming that a negligible amount of time is spent roosting away from the nest, the time spent away from the nest was considered to be a reliable proxy of foraging trip duration, which is an important component of daily energy expenditure^4,5^. However, to avoid biases from adults resting at the colony (but not visible in the video), only trips over 10 min were used, to exclude periods when birds may have left the nest for reasons other than foraging (human disturbance or predator avoidance)^6^. We found no significant differences in trip durations and feeding rates between the two colonies (permutation test, all p values > 0.1); thus all data were pooled for further analysis.

Sensitivity analyses of time-energy-budget models were run to assess the impact of different parameters on the estimated energy budget. The variation (expressed in %) of the model output (daily food intake) was calculated one parameter at the time by substituting the mean value of the parameter by the mean ± SD^7^. In order to calculate the range of variation for daily food intake, time at the colony was increased or decreased in response to variation in daily time spent flying or resting at the colony. The maximum variation of the model was calculated for the most demanding condition (using mean + SD of time-activity budget parameters and body mass and mean ‒ SD of assimilation efficiency and calorific value of prey). The minimum variation of the model was calculated for the least demanding condition (using mean ‒ SD of time-activity budget parameters and body mass and mean + SD of assimilation efficiency and calorific value of prey).

**Influence of diet composition on modelled chick daily food intake (DFI):** One of our aims was to compare our observations to our model estimates of chick daily food intake (DFI). To estimate the DFI of chicks from our empirical observations, we used a photo-sampling technique that allowed an accurate determination of fish species and, for anchovy only, allowed us to accurately and precisely estimate fish standard length^8^. From these lengths – and by calculating length-weight regressions for dead anchovy sampled during pelagic recruit surveys (Table S1-3) – we were able to convert estimated fish lengths to fish mass and so estimate the DFI of chicks, or more accurately, the daily anchovy intake. To make our model estimate comparable to that derived from the empirical data described above, we assume that anchovy makes up the entire diet when modelling chick DFI and model the energy content of each prey item as 6.22 ± 0.65 kJ·g^−1^ based on the wet mass for anchovy (see equation 3 in the main text). However, in reality anchovy makes up ~65% of prey items returned to the colony by provisioning greater crested terns^9^, other forage fish make up another 29% and other fish another 2%. A reasonable approximation of the average energy content of the diet could therefore use 6.22 ± 0.65 kJ·g^−1^ for anchovy (65% of the diet), 5.65 kJ·g^−1^ for horse mackerel (2% of the diet), 6.59 kJ·g^−1^ for sardine (2% of the diet) and 5.91 kJ·g^−1^, the average value for teleost fish in this ecosystem^10^ for other fish (31% of the diet). The mean calorific value of prey then becomes (6.22 × 0.65) + (5.91 × 0.31) + (6.59 × 0.02) + (5.65 × 0.02) = 6.12 kJ·g^−1^. Rerunning our model using 6.12 kJ·g^−1^ rather than 6.22 ± 0.65 kJ·g^−1^ resulted in chick DFI of 76.3 g·d^−1^ instead of the original 75.6 g·d^−1^ – a change of just 0.9%. We therefore retained the results using only anchovy (and 6.22 ± 0.65 kJ·g^−1^) to ensure comparability with our empirical observations.

**References**

1. Heydorn, M. J. & Williams, A. J. Swift Terns: observations at Possession Island in 1988. *Bontebok,* **8**, 26–27 (1993).

2. Hall, C. S., Kress, S. W., & Griffin, C. R. Composition, spatial and temporal variation of Common and Arctic Tern chick diets in the Gulf of Maine. *Waterbirds*, 430–439 (2000).

3. Nicholson, L. Breeding strategies and community structure in an assemblage of tropical seabirds on the Lowendal Islands, Western Australia. Unpubl. Doctoral dissertation, Murdoch University, Perth (2002).

4. Fyhn, M., Gabrielsen, G. W., Nordøy, E. S., Moe, B., Langseth, I. & Bech, C. Individual variation in field metabolic rate of kittiwakes (*Rissa tridactyla*) during the chick-rearing period. *Physiological and Biochemical Zoology*, **74**, 343–355 (2001).

5. Rishworth, G. M., Tremblay, Y. & Green, D. B. Drivers of time-activity budget variability during breeding in a pelagic seabird. *PLoS ONE*, **9**, 1–17 (2014).

6. McLeay, L. J., Page, B., Goldsworthy, S. D., Paton, D. C., Teixeira, C., Burch, P., & Ward, T. Foraging behaviour and habitat use of a short-ranging seabird, the crested tern. *Marine Ecology Progress Series,* **411**, 271−283 (2010).

7. Enstipp, M. R. *et al.* Foraging energetics of North Sea birds confronted with fluctuating prey availability. In: Boyd, S.; Wanless, S., Camphuysen, C. J., (eds.) Top predators in marine ecosystems: their role in monitoring and management. Cambridge University Press, Cambridge UK, 191–210 (2006).

8. Gaglio, D., Cook, T. R., Connan, M., Ryan, P. G. & Sherley, R. B. Dietary studies in birds: testing a non-invasive method using digital photography in seabirds. *Method in Ecology and Evolution.***8,** 214–222 (2017).

9. Gaglio, D., Cook, T.R., McInnes, A., Sherley, R.B., & Ryan, P.G. Foraging plasticity in seabirds: a non-invasive study of the diet of greater crested terns breeding in the Benguela Region. *PLoS ONE*, **13**, e0190444 (2018).

10. Balmelli, W., & Wickens, P. A. Estimates of daily ration for the South African (Cape) fur seal. *African Journal of Marine Science,* **14**, 151–157 (1994).

11. Visser, G. H. Chick growth and developments in seabirds. In Biology of Marine Birds (eds: Schreiber EA, Burger J). CRC Press, 439–465 (Boca Raton, 2002).

12. Crawford, R. J. M. *et al.* Longevity, inter-colony movements and breeding of Crested Terns in South Africa. *Emu*, **102**, 1–9 (2002).


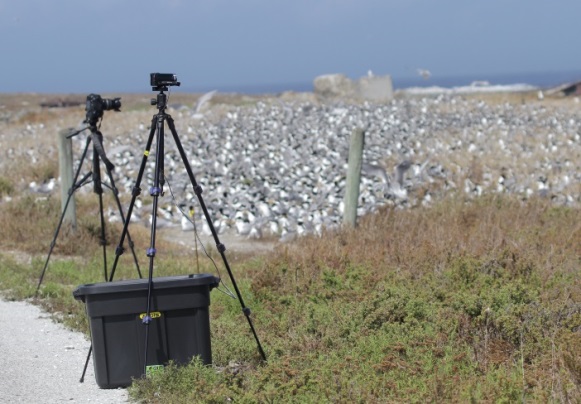

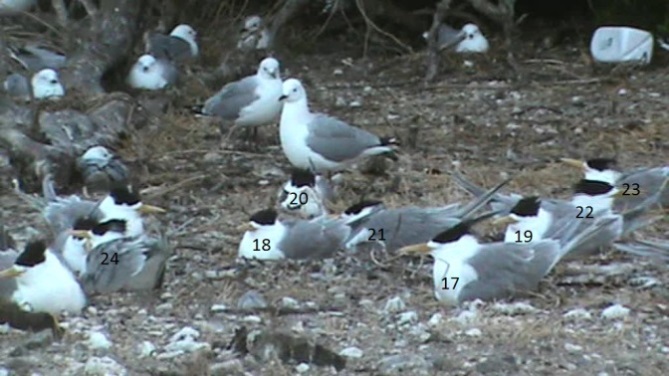


**Figure S1**: *Left*: Illustration of the setup of video-cameras near a greater crested tern colony. *Right*: Snapshot from a video monitoring nest-cup activities of greater crested terns (Hartlaub’s gulls *Chroicocephalus hartlaubii* are breeding in the background). Numbers indicate ID of nests monitored.


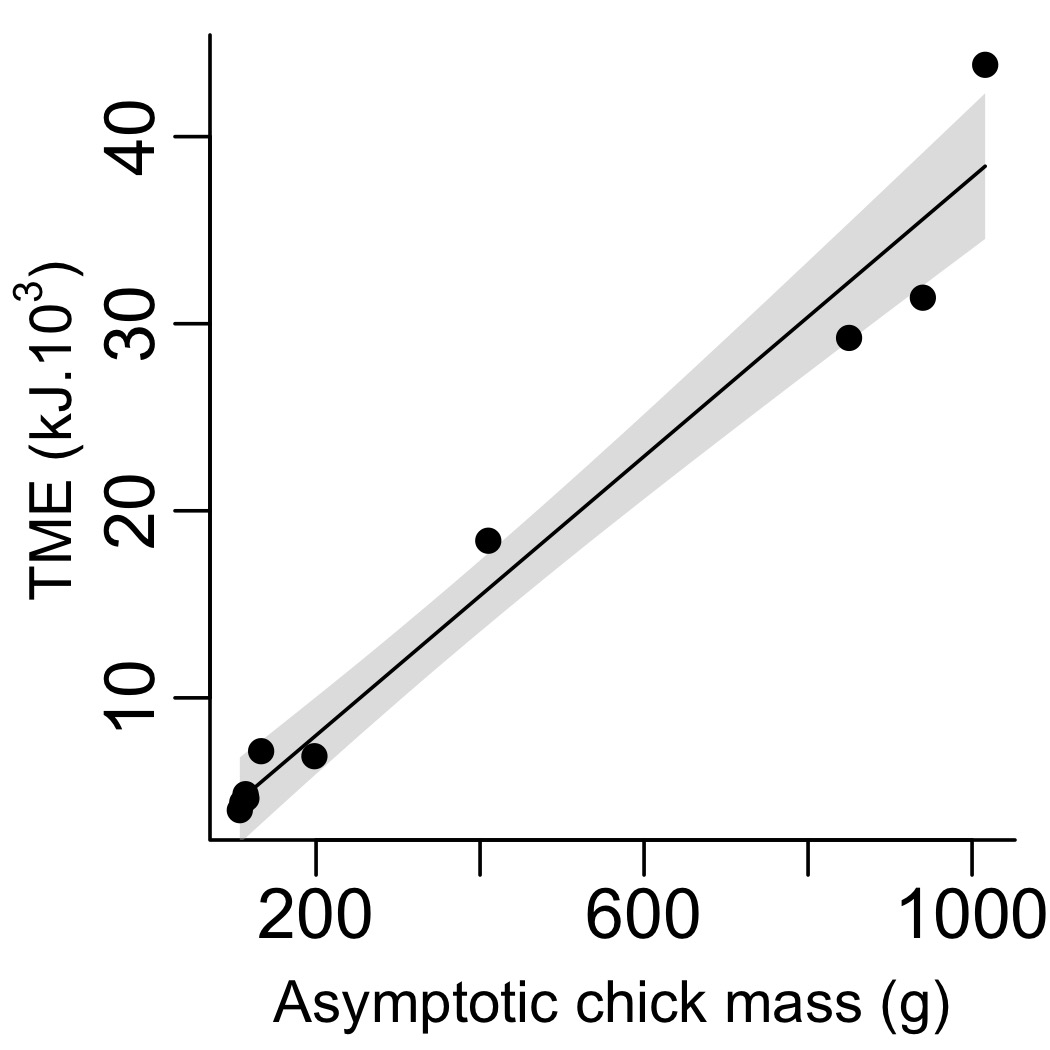


**Figure S2**: The allometric regression fit to published data on the total amount of energy metabolized until fledging (*TME*) and asymptotic chick mass (*A*) for 10 larid species^36^ (see equation 3, main text).


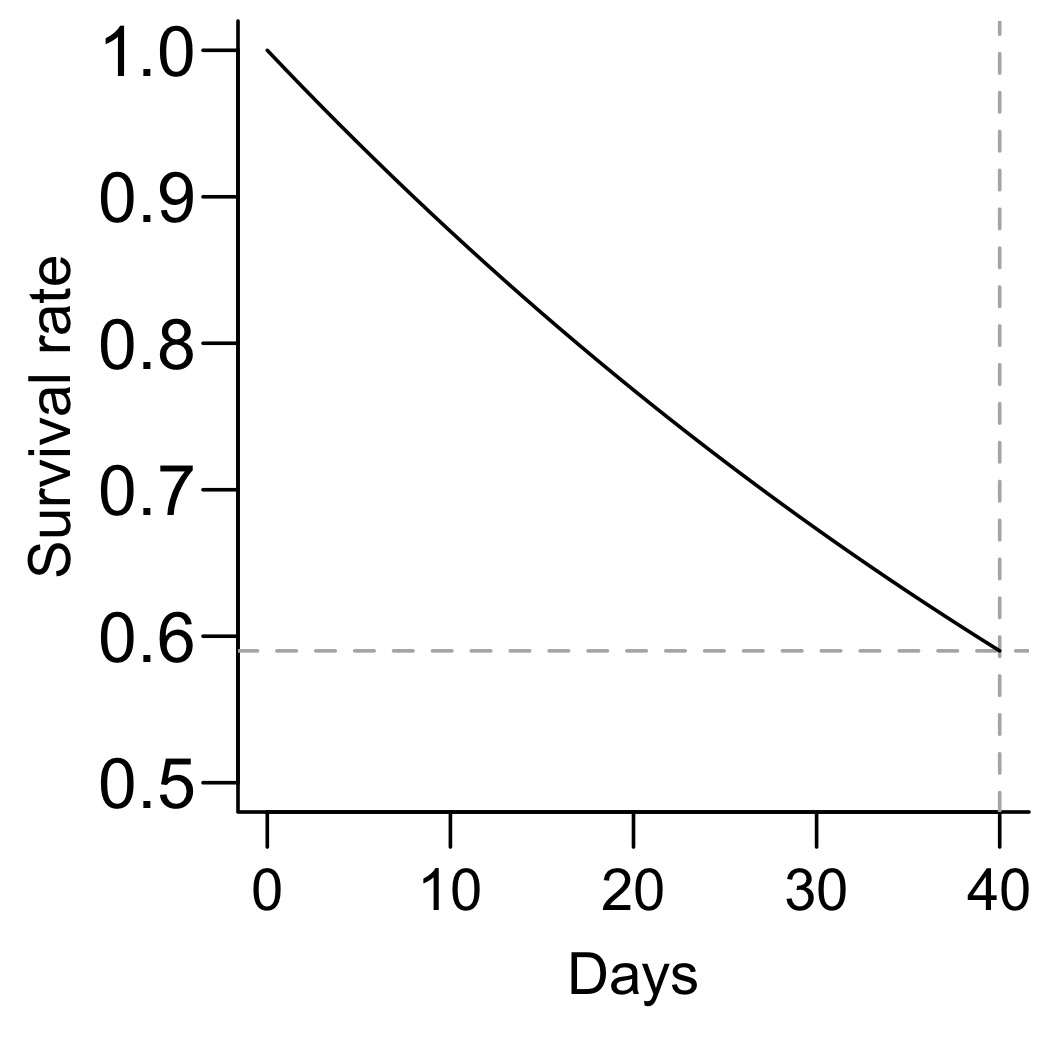


**Figure S3**: The survival function (black line) for greater crested tern chicks used in the bioenergetics model. The curve is based on an observed breeding success of 0.59 chicks fledged per pair (shown by the horizontal dashed line), a single egg clutch and a 40-day fledging period^12^ (shown by the vertical dashed line), yielding a daily mortality rate of log(0.59)/40 (see equation 5, main text).

**Table S1**: Life-history traits of four seabird species breeding in the Benguela system (data from Crawford *et al.* 2005).

| **Trait** | **Greater crested tern** | **African penguin** | **Cape gannet** | **Cape cormorant** |
| --- | --- | --- | --- | --- |
| Brood size | 1 | 2 | 1 | 2.5 |
| Foraging range (km) | ca 10 | ca 40 | ca 200 | ca 40 |
| Foraging techniques | Several* | Pursuit diving | Plunging, scavenging | Pursuit diving |
| Fidelity to colony | N | Y | Y | N |
| Post-fledging care | Y** | N | N | Y |
| Breeding period (days) | 68 days | 115 days | 141 days | 84 days |

* Surface-Seizing, dipping, plunge-diving, scavenging , kleptoparasitism, diving from perches; ** up to 4 months.

**Table S2**: Sensitivity analysis (variation of the model output expressed in %) of energy-budget models of greater crested terns (see Supplementary information SI-1for details).

| **Parameter** | **Incubation** | **Early provisioning** | **Late provisioning** |
| --- | --- | --- | --- |
| Body mass | ± 8.1 | ± 8.1 | ± 8.1 |
| Time flying | ± 1.7 | ± 1.8 | ± 3.5 |
| Time diving | ± 0.0006 | ± 0.002 | ± 0.0005 |
| Assimilation efficiency | ± 4.4 | ± 4.4 | ± 4.4 |
| Calorific value of prey | ± 19.7 | ± 19.7 | ± 19.8 |
| Maximum variation | + 38.6 | + 38.5 | + 42.3 |
| Minimum variation | ‒ 23.2 | ‒ 23.2 | ‒ 25.2 |

**Table S3**: Length-weight regressions for anchovy sampled during pelagic recruit surveys (May/June 2013–2015). Data from the Department of Agriculture, Forestry and Fisheries.

| **Year** | **WBM** | **r^2^** | **n** |
| --- | --- | --- | --- |
| 2013 | 0.0050*CL^3.2438^ | 0.976 | 2,560 |
| 2014 | 0.0072*CL^3.0834^ | 0.934 | 2,096 |
| 2015 | 0.0061*CL^3.1618^ | 0.975 | 3,618 |

WBM = aCL^b^, where WBM is in g and CL in cm.

Supplementary Information S2

**JAGS code used to specify the bioenergetics model:**

model {

############ Priors for Time energy budget model ############

weight ~ dnorm(m.bm, tau.wse) # Prior for mean and SD mass of swift terns

tau.wse <- pow(sd.bm, -2)

water ~ dnorm(m.tw, tau.tw) # Prior for time spent on the water

tau.tw <- pow(sd.tw, -2)

anch ~ dnorm(m.acca, tau.acca) # Prior for mean and sd energy content of anchovy prey

tau.acca <- pow(sd.acca, -2)

asseff ~ dnorm(m.ae, tau.ae) # Prior for mean and sd of assimulation efficiency

tau.ae <- pow(sd.ae, -2)

############################################################

############ Priors for allometric equation ############

alpha ~ dnorm(0, 0.0000001) # Non-informative normal prior for intercept for allometric equation on Larid data

beta ~ dnorm(0, 0.0000001) # Non-informative normal prior for slope for allometric equation on Larid data

sigma ~ dunif(1500, 4500)

tau.resid <- pow(sigma, -2)

############ Likelihood for Allometric regression ############

for (i in 1:n) # for each of n species

{

TME[i] ~ dnorm(mu[i], tau.resid) ## allometric equation on Larid data

mu[i] <- alpha + beta * Ass[i]

}

############################################################

############ Priors for feeding rate by breeding stage (gamma regression, log-link) ############

for(i in 1:max(bs.Fr)){

alpha.Fr[i] ~ dnorm(0, 0.0000001) # Non-informative normal prior for intercept for Feeding rate estimate by breeding stage

}

shape.Fr ~ dunif(0, 100)

############ Likelihood for feeding rate by breeding stage ############

for (i in 1:m) # for each of m feeding rate data points

{

lin.pred.Fr[i] <- alpha.Fr[bs.Fr[i]]

Fr[i] ~ dgamma(shape.Fr, shape.Fr/exp(lin.pred.Fr[i]))

}

############################################################

############ Priors for Anchovy mass by breeding stage (gamma regression, log-link) ############

for(i in 1:max(bs.Am)){

alpha.Am[i] ~ dnorm(0, 0.0000001) # Non-informative normal priors for intercept for Anchovy mass estimate by breeding stage

}

shape.Am ~ dunif(0, 100)

############ Likelihood for Anchovy mass by breeding stage ############

for (i in 1:p) # for each of p anchovy mass data points

{

lin.pred.Am[i] <- alpha.Am[bs.Am[i]]

Am[i] ~ dgamma(shape.Am, shape.Am/exp(lin.pred.Am[i]))

}

############################################################

############ Priors for Trip duration by breeding stage (gamma regression, log-link) ############

for(i in 1:max(bs.Td)){

alpha.Td[i] ~ dnorm(0, 0.0000001)

}

shape.Td ~ dunif(0, 100)

############ Likelihood for Trip duration by breeding stage ############

for (i in 1:q) {

lin.pred[i] <- alpha.Td[bs.Td[i]]

# dgamma(shape, rate) in JAGS:

Td[i] ~ dgamma(shape.Td, shape.Td/exp(lin.pred[i]))

}

############################################################

############ Priors for number of trips by breeding stage (gamma regression, log-link) ############

for(i in 1:max(bs.Nt)){

alpha.Nt[i] ~ dnorm(0, 0.0000001)

}

shape.Nt ~ dunif(0, 100)

############ Likelihood for number of trips by breeding stage ############

for (i in 1:s) {

lin.pred.Nt[i] <- alpha.Nt[bs.Nt[i]]

# dgamma(shape, rate) in JAGS:

Nt[i] ~ dgamma(shape.Nt, shape.Nt/exp(lin.pred.Nt[i]))

}

############################################################

############ Derived parameters ############

MCME <- ((beta*acm)+alpha)/dtf # Mean chick metabolisable energy from n = 10 tern and gull (Laridae) species

FR.e <- exp(alpha.Fr[1])-0.01 # Observed feeding rate during early chick rearing

FR.l <- exp(alpha.Fr[2])-0.01 # Observed feeding rate during late chick rearing

AM.e <- exp(alpha.Am[1]) # Observed anchovy mass fed to chicks during early chick rearing

AM.l <- exp(alpha.Am[2]) # Observed anchovy mass fed to chicks during late chick rearing

OCDFI.e <- FR.e*AM.e # Observed chick daily food intake during early chick rearing

OCDFI.l <- FR.l*AM.l # Observed chick daily food intake during late chick rearing

TD.i <- exp(alpha.Td[1]) # Observed foraging trip duration during incubation

TD.e <- exp(alpha.Td[2]) # Observed foraging trip duration during early chick rearing

TD.l <- exp(alpha.Td[3]) # Observed foraging trip duration during late chick rearing

NT.i <- exp(alpha.Nt[1]) # Observed number of foraging trips during incubation

NT.e <- exp(alpha.Nt[2]) # Observed number of foraging trips early chick rearing

NT.l <- exp(alpha.Nt[3]) # Observed number of foraging trips late chick rearing

fly <- TD.i*NT.i

fly.e <- TD.e*NT.e

fly.l <- TD.l*NT.l

rest <- 24-fly

rest.e <- 24-fly.e

rest.l<- 24-fly.l

############ Time-energy budget model ############

COSTR <- ((BMR*2)*weight)*((rest*60)*60)/1000

COSTF <- ((BMR*5.2)*weight)*((fly*60)*60)/1000

FMR <- ((((DEE*1000)/86400)/BMR/weight)) # Field metabolic rate

ADFI <- DEE/(anch*(asseff/100)) # Adult daily food intake incubation

CPUE <- ADFI/((fly*60)+water) # Catch per unit effort during incubation

CDFI <- MCME/(anch*(asseff/100))

ADFI.e <- DEE.e/(anch*(asseff/100)) # Adult daily food intake early chick rearing

COSTR.e <- ((BMR*2)*weight)*((rest.e*60)*60)/1000

COSTF.e <- ((BMR*5.2)*weight)*((fly.e*60)*60)/1000

FMR.e <- ((((DEE.e*1000)/86400)/BMR/weight)) # Field metabolic rate

CPUE.e <- ADFI.e/((fly.e*60)+water) # Catch per unit effort during early chick rearing

ADFI.l <- DEE.l/(anch*(asseff/100)) # Adult daily food intake late chick rearing

COSTR.l <- ((BMR*2)*weight)*((rest.l*60)*60)/1000

COSTF.l <- ((BMR*5.2)*weight)*((fly.l*60)*60)/1000

FMR.l <- ((((DEE.l*1000)/86400)/BMR/weight)) # Field metabolic rate

CPUE.l <- ADFI.l/((fly.l*60)+water) # Catch per unit effort during late chick rearing

DEE <- COSTR+COSTF # Daily energy expenditure during incubation

DEE.e <- COSTR.e+COSTF.e # Daily energy expenditure during early chick rearing

DEE.l <- COSTR.l+COSTF.l # Daily energy expenditure during late chick rearing

CMR <- log(cfpp)/dtf # Chick daily mortality rate (assuming a survival rate of 0.59 over 40 days)

for (i in 1:dtf) {

CSF[i] <- exp(CMR*i) # chick survival function

CDFI.P[i] <- (CDFI*CSF[i])/2 # chick daily food intake delivered per parent

}

m.CDFI.P <- mean(CDFI.P)

TDFI.e <- ADFI.e + m.CDFI.P # Total Daily Food intake during early chick rearing

TDFI.l <- ADFI.l + m.CDFI.P # Total Daily Food intake during late chick rearing

TDFI.prov <- (TDFI.e*0.1)+(TDFI.l*0.9) # The mean TDFI for the provisioning period, weighted by length of the two periods

##### Predictions for the allometric equation plot:

for (i in 1:50) #

{

Pred[i] <- alpha + beta*n.dat[i]

}

}
